# A primate model of severe malarial anaemia: a comparative pathogenesis study

**DOI:** 10.1038/s41598-019-55377-3

**Published:** 2019-12-12

**Authors:** Amber I. Raja, Elizabeth B. Brickley, Jessica Taaffe, Timmy Ton, Zhen Zhao, Kevin W. Bock, Sachy Orr-Gonzalez, Marvin L. Thomas, Lynn E. Lambert, Ian N. Moore, Patrick E. Duffy

**Affiliations:** 1 Laboratory of Malaria Immunology and Vaccinology, National Institute of Allergy and Infectious Diseases, National Institutes of Health, Bethesda, Maryland United States of America; 2 Department of Infectious Disease Epidemiology, London School of Hygiene & Tropical Medicine, London, United Kingdom; 3 Department of Laboratory Medicine, Clinical Center, National Institutes of Health, Bethesda, Maryland United States of America; 4 Weill Cornell Medicine, New York City, New York United States of America; 5 Comparative Medicine Branch, Infectious Disease Pathogenesis Section, National Institute of Allergy and Infectious Diseases, National Institutes of Health, Rockville, Maryland United States of America; 6 Division of Veterinary Resources, Office of Research Services, National Institutes of Health, Bethesda, Maryland United States of America

**Keywords:** Parasite host response, Parasite biology

## Abstract

Severe malarial anaemia (SMA) is the most common life-threatening complication of *Plasmodium falciparum* infection in African children. SMA is characterised by haemolysis and inadequate erythropoiesis, and is associated with dysregulated inflammatory responses and reduced complement regulatory protein levels (including CD35). However, a deeper mechanistic understanding of the pathogenesis requires improved animal models. In this comparative study of two closely related macaque species, we interrogated potential causal factors for their differential and temporal relationships to onset of SMA. We found that rhesus macaques inoculated with blood-stage *Plasmodium coatneyi* developed SMA within 2 weeks, with no other severe outcomes, whereas infected cynomolgus macaques experienced only mild/ moderate anaemia. The abrupt drop in haematocrit in rhesus was accompanied by consumption of haptoglobin (haemolysis) and poor reticulocyte production. Rhesus developed a greater inflammatory response than cynomolgus macaques, and had lower baseline levels of CD35 on red blood cells (RBCs) leading to a significant reduction in the proportion of CD35^+^ RBCs during infection. Overall, severe anaemia in rhesus macaques infected with *P. coatneyi* has similar features to SMA in children. Our comparisons are consistent with an association of low baseline CD35 levels on RBCs and of early inflammatory responses with the pathogenesis of SMA.

## Introduction

The burden of malaria is greatest amongst children under the age of 5 years, who suffer 70% of all malaria related-deaths and have the highest rates of severe malaria including severe malarial anaemia (SMA)^1^. SMA is defined as a haemoglobin less than 5 g/dL (haematocrit less than 15%) in parasitaemic children and a haemoglobin less than 7 g/dL (haematocrit less than 20%) in parasitaemic adults^2^. SMA has been estimated to account for 54% of malaria-related deaths in areas of holoendemic malaria transmission^3^. Haemolysis and dyserythropoiesis are key features of SMA^4–9^, but the causal haematological and immune mediators of this fatal condition are not well defined. Inflammatory cytokine levels in plasma as well as complement regulatory protein levels on red blood cells (RBCs) differ between children presenting with SMA versus those with mild malaria [reviewed in^10–13^], but these associations do not confirm causality.

Animal models are essential to interrogate the causation of specific malarial outcomes. *Plasmodium coatneyi* (*P. coatneyi*) infects non-human primates and has phenotypical similarities to *Plasmodium falciparum* (*P. falciparum*), with a tertian periodicity (i.e. ~48 hour blood-stage life cycle) and an ability to rosette and sequester in deep vascular beds^14–20^. *P. coatneyi* can infect several non-human primates, with varying degrees of severity^21–23^. For instance, blood-stage *P. coatneyi* infection in rhesus and Japanese macaques causes severe malaria, variously characterised as cerebral malaria, SMA or multisystem dysfunction^14,16,21,22,24,25^, whereas in cynomolgus, Southern pig-tailed and stump-tailed macaques it causes mild to moderate disease [^14,23^; reviewed in^26^].

Here, we compare rhesus and cynomolgus macaques for their differing susceptibility to develop SMA during *P. coatneyi* blood-stage infection and characterise the anaemia by features such as time of onset, haemolysis, and reticulocyte production. We interrogate potential causal factors, including complement regulatory proteins, immune activation, and cytokine/ chemokine profiles, for their differential expression in the two models and their temporal relationship to the onset of SMA.

## Methods

### Ethics statement

All animal work was approved by the Animal Care and Use Committee at the National Institutes of Health under approval LMIV 9E and Animal Assurance number NIH IRP A4149-01. The study was carried out under National Institute of Allergy and Infectious Disease (NIAID) Division of Intramural Research (DIR) Animal Care and Use Committee guidelines. Parasite inoculations and blood collections on days 0, 4, 7, and daily from day 9 onward were carried out under anaesthesia and all efforts were made to minimise the suffering of study animals.

### Monkeys

Female rhesus macaques (*Macaca mulatta*) of Indian origin aged 4 to 6 years old and female cynomolgus macaques (*Macaca fascicularis*) of Vietnamese origin aged 2 to 12 years old were obtained from National Institutes of Health breeding colonies. Monkeys were housed in an American Association for the Accreditation of Laboratory Animal Care (AAALAC)-approved facility, with a dedicated American College of Laboratory Animal Medicine (ACLAM)-accredited staff veterinarian.

Animals were immunised against measles and tested for *Mycobacterium tuberculosis* exposure quarterly. Animal observation was performed at least twice-daily and abnormalities were reported to the veterinarian. Standard National Institute of Allergy and Infectious Disease enrichment operating procedures were followed, and monkeys were housed in AAALAC-approved cages. A high fibre monkey diet was provided, supplemented by fresh fruits, vegetables and nuts, with water ad libitum. Animals were provided with hanging and floor toys, with perches in the cages allowing for exercise. The facility was maintained at a temperature between 73°F and 79°F, a humidity of less than 70%, and a regular light cycle.

### Malaria parasite

Blood-stage *Plasmodium coatneyi* (Hackeri strain) parasites originating from a cryopreserved stock from the Centers for Disease Control and Prevention, were blood-passaged once through a spleen-intact rhesus macaque, and then stored frozen at a parasitaemia of 7.05% in 0.7 mL aliquots.

### Blood-stage *P. coatneyi* infection and monitoring of disease

**Figure 1:**
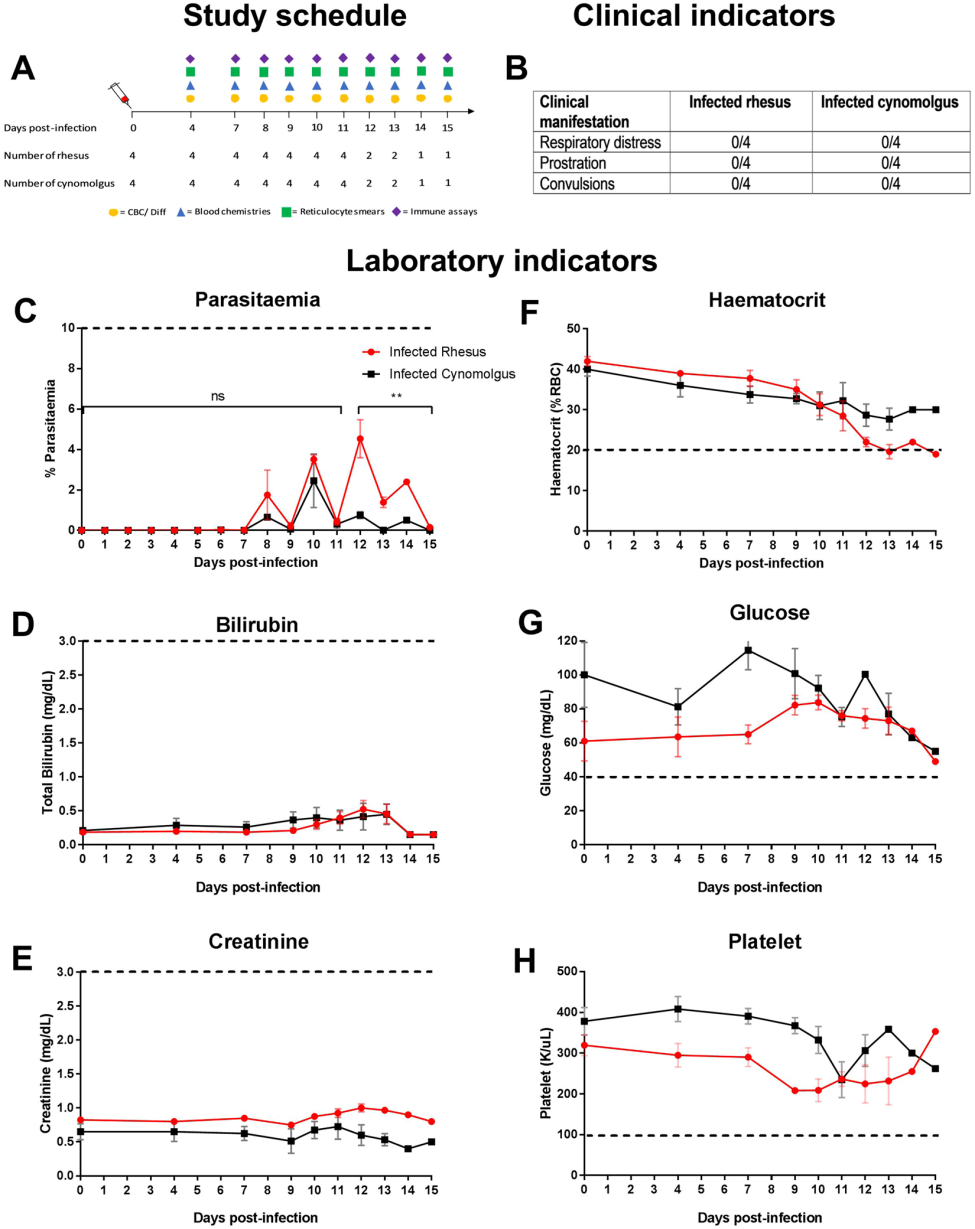
Manifestation of severe malaria during blood-stage *P. coatneyi* infection in rhesus and cynomolgus macaques. (**A**) The study schedule indicates sample collection timepoints and the number of animals per species during the course the infection. (**B**) Clinical indicators of severe malaria and (**C**–**H**) laboratory indicators of severe malaria in rhesus (n = 4) and cynomolgus (n = 4) macaques infected with blood-stage *P. coatneyi*. For the graphs in the left panel (**C**–**E**) values above the dashed line represent severe malaria and for the graphs in the right panel values below the dashed line represent severe malaria. Error bars show the SEM for each group. Using linear mixed-effects models the (**C**) parasitaemias of infected rhesus and cynomolgus macaques were compared between days 4 and 11, when all animals remained alive (p = 0.56), and days 12 and 15, when the number of animals per group was decreasing (**p < 0.001).

Following anaesthesia, four rhesus and four cynomolgus macaques were injected intravenously through the saphenous vein with freshly thawed *P. coatneyi* infected RBCs. Each rhesus macaque was paired with a cynomolgus macaque based on weight. Blood films for reticulocyte counts were collected starting at baseline and prepared using New Methylene Blue stain (Jorgensen Laboratories, Inc.) according to the manufacturer’s instructions. Reticulocyte counts were determined by examining a total of 2,000 RBCs, and reticulocyte production indices were calculated as previously described^27^. Following infection, parasitaemia was monitored daily by examining Giemsa-stained thin blood films and calculated using microscopic examination of 2,000 to 10,000 RBCs. Animals were also monitored using complete blood counts and differentials and blood chemistries on days 0, 4, 7 and daily from day 9 onward (Fig. 1A).

Paired animals were euthanised using intravenous infusion of B-euthanasia™ (Pentobarbital Sodium) at 0.2 mL/kg when one animal in the pair reached any of the pre-determined severe malaria criteria (Supplementary Table S1), and tissue samples were collected.

### Assessment of complement regulatory protein on the surface of RBCs

Blood was collected from animals into Lithium-heparin tubes. Two microliters of blood were added to 100 µL of PBS, and an antibody mastermix containing anti-CD45 (D058-1283, V500; BD Horizon), anti-CD35 (E11, FITC; Biolegend) and anti-CD71 (L01.1, APC; BD Bioscience) was added to each sample. Samples were incubated at 4 °C for 30 min and washed three times in PBS by centrifugation at 314 × *g* for 5 min. The cells were then resuspended in 3 mL of PBS, and immediately analysed using the LSR II flow cytometer (BD Biosciences), FACSDiva software version 8.0.1 (BD Biosciences), and FlowJo software version 10 (FlowJo, LLC). The gating strategy in Supplementary Fig. S1 was used to identify markers on RBCs.

### Assessment of T cell activation in peripheral blood

Blood was collected into Lithium-heparin tubes. An antibody mastermix containing anti-CD3 (SP34- 2, AF700; BD Pharmingen), anti-CD4 (L200, APC-H7; BD Pharmingen), anti-CD8 (SK1, PerCP; Biolegend) and anti-CD69 (FN50, PE-Cy7; eBioscience) was added to 100 µL of blood. Samples were incubated for 30 min at 4 °C, and washed three times in PBS by centrifugation at 314 × *g* for 5 min. The cells were resuspended in 400 µL of PBS, and immediately analysed using the LSR II flow cytometer (BD Biosciences), FACSDiva software version 8.0.1 (BD Biosciences), and FlowJo software version 10 (FlowJo, LLC). The gating strategy in Supplementary Fig. S2 was used to identify activated T cells.

### Assessment of plasma cytokines and chemokines

Blood was collected into K2 EDTA tubes and centrifuged within 30 min of collection at 1,000 × *g* for 10 min. The plasma was removed and stored at −80 °C.

Interleukin-1β (IL-1β), IL-4, IL-5, IL-6, IL-8, IL-10, IL-12p40, IL-23, interferon gamma (IFN-γ), tumor necrosis factor alpha (TNF-α), monocyte chemoattractant protein 1 (MCP-1), interferon gamma-induced protein 10 (IP-10), granulocyte-macrophage colony-stimulating factor (GM-CSF), granulocyte-colony stimulating factor (G-CSF), macrophage inflammatory protein 1 alpha (MIP-1α), macrophage inflammatory protein 1 beta (MIP-1β), RANTES, eotaxin, and erythropoietin (EPO) were measured using LEGENDplex custom kits (Biolegend) according to the manufacturer’s instructions. Biomarker levels were quantified using LEGENDplex^TM^ Data Analysis Software.

### Histology

Following euthanasia, tissue samples collected from various organs were fixed in 10% Neutral Buffered Formalin for at least 72 hours before being transferred to 70% ethanol for long-term storage. Processing into paraffin blocks, as well as staining with Haematoxylin & Eosin (H&E) and Perls’ Prussian Blue, was performed by Histoserv, Inc. (Germantown, MD). Accumulation of mature parasites in tissue was assessed in H&E stained slides, and was evident when numerous small calibre vessels were completely filled with RBCs, at times in a Rouleaux-like formation, that consistently contained black pigmented foci, indicating mature parasite stages.

### Immunohistochemistry

Formalin-fixed paraffin-embedded sections were cut at 5 µm depth and transferred to positively-charged microscope slides. Immunohistochemical staining using rabbit polyclonal anti-Iba1 (Wako Chemicals USA, Inc.) and rabbit polyclonal anti-Myeloperoxidase (Abcam) was carried out on the Bond RX (Leica Biosystems) platform according to manufacturer-supplied protocols. Briefly, sections were deparaffinised and rehydrated. Heat-induced epitope retrieval (HIER) was performed using Epitope Retrieval Solution 1, pH 6.0, heated to 100 °C for 20 min. The primary antibodies were applied at 1:800 (Iba1) and 1:30 (Myeloperoxidase) dilutions. Detection with Fast Red chromogen was completed using the Bond Polymer Refine Red Detection kit (Leica Biosystems #DS9390), a biotin-free polymeric alkaline phosphatase (AP)-linked antibody conjugate system. Slides were counterstained with haematoxylin, and then cleared through gradient alcohol and xylene washes prior to mounting and coverslipping. All specimens were examined by light microscopy using an Olympus BX51 microscope and photomicrographs were taken using an Olympus DP73 camera.

### Statistical analysis

Positively skewed continuous variables were log_10_-transformed before analysis. Prior to infection (i.e. at day 0) and at the time of necropsy, geometric mean biomarker levels were compared cross-sectionally between the rhesus and cynomolgus macaques using an independent two sample t-test. After infection (i.e. between days 4 and 11), mean biomarker levels were compared between species using linear mixed-effects models that adjusted for linear time trends and day 0 biomarker levels and allowed for individual-specific random effects. Parasitaemia levels were also compared between species using linear mixed-effects models that adjusted for linear time trends and allowed for individual-specific random effects during two time periods: days 4 to 11 post-infection (i.e. when all animals were live) and days 12 to 15 (i.e. when the number of animals followed was decreasing). All p-values are from two-sided tests, and all analyses were performed using Stata version 15 (StataCorp LP, College Station, TX, USA), R version 3.2.5 or GraphPad Prism version 7.

## Results

### Comparative primate models of malarial anaemia

Rhesus and cynomolgus macaques infected with blood-stage *P. coatneyi* were monitored for signs of severe malaria (Supplementary Table S1). These animals did not suffer from respiratory distress, prostration or convulsions (Fig. 1B). In addition, neither species suffered from hyperparasitaemia (parasitaemia >10%), jaundice (bilirubin >3 mg/dL), renal impairment (creatinine >3 mg/dL), hypoglycaemia (glucose <40 mg/dL) or thrombocytopenia (platelets <100 K/µL) (Fig. 1C–E,G,H).

The only severe manifestation of malaria was SMA, which was observed in all four *P. coatneyi*-infected rhesus macaques, but not in infected cynomolgus macaques (Fig. 1F). Although infected macaques of both species exhibited a gradual decline in their haematocrit during infection, rhesus macaques’ haematocrit reached an inflection point starting between day 7 and 9. Thereafter, all infected rhesus macaques developed SMA, defined as a haematocrit of <20% (as all rhesus macaques were adults), ranging from 17 to 19%, between days 11 and 15 post-infection. Infected cynomolgus macaques, on the other hand, had a steady and modest decline in their haematocrit and did not develop SMA (haematocrit ranged between 24 to 30%) in the 15-day timeframe. In both macaque species, *P. coatneyi* was synchronous, with a tertian periodicity (Fig. 1C) indicating mature stage parasite sequestration within deep vascular beds, and prior to day 12, rhesus and cynomolgus macaques supported similar parasite densities (p = 0.313).

### Deep tissue parasite accumulation and haemozoin deposition

**Figure 2:**
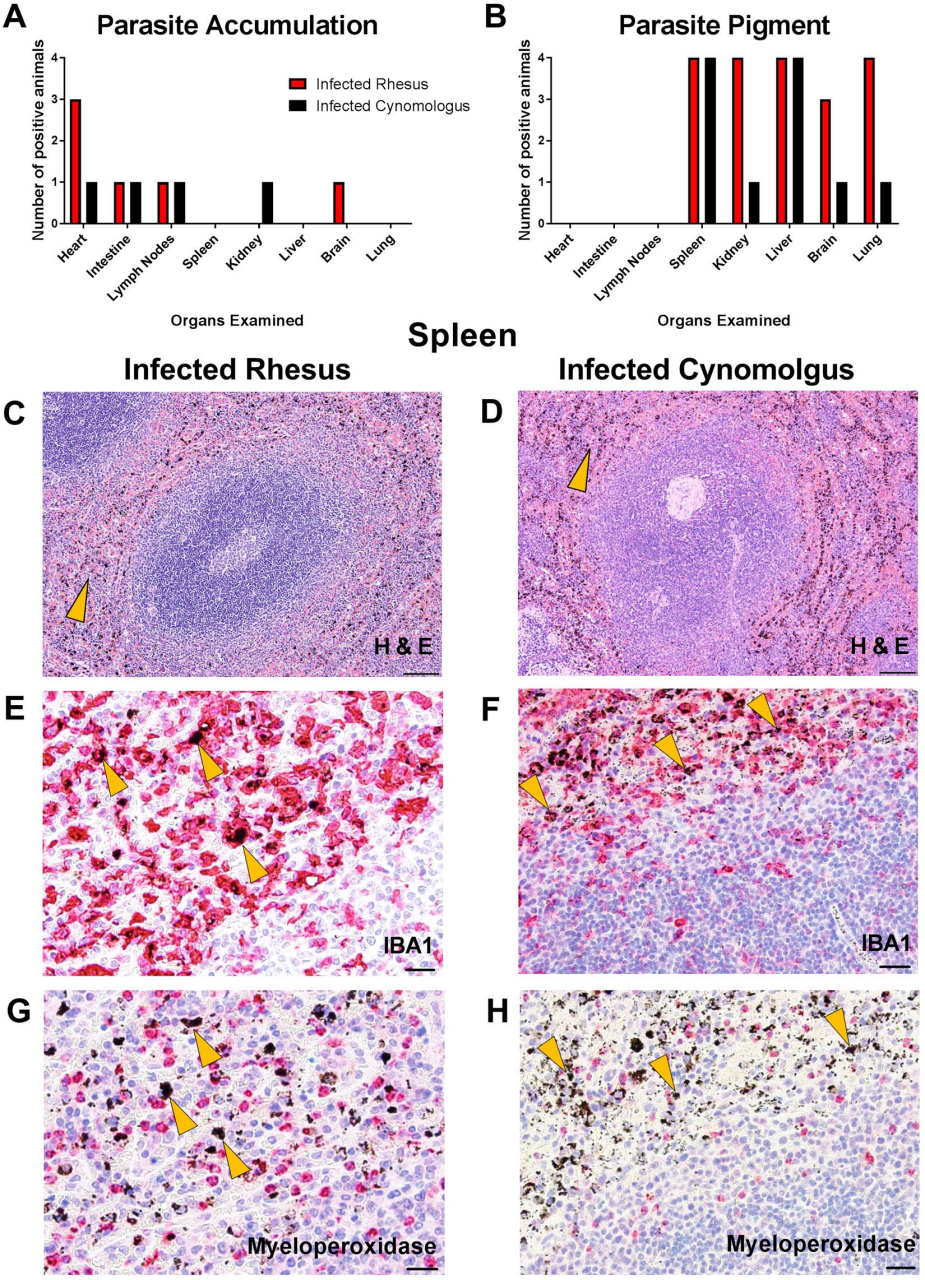
Deep tissue parasite accumulation and haemozoin deposition patterns of *P. coatneyi* in infected rhesus and cynomolgus macaques. (**A**) Mature parasite accumulation and (**B**) deposition of parasite pigment was assessed using H&E staining of organs from infected rhesus and cynomolgus macaques. H&E stained spleens from one representative (**C**) rhesus and (**D**) cynomolgus macaque. IBA1 stained spleens from one representative (**E**) rhesus and (**F**) cynomolgus macaque to assess whether parasite pigment was within the cytoplasm of macrophages. Myeloperoxidase stained spleens from one representative (**G**) rhesus and (**H**) cynomolgus macaque to assess association of parasite pigment and neutrophils. Arrowheads indicate areas of haemozoin pigment. Images (**C**) and (**D**) were taken at 4X magnification (scale bars represent 200 µm), (**E**) and (**G**) were taken at 40X magnification (scale bars represent 20 µm), and (**F**) and (**H**) were taken at 20X magnification (scale bars represent 50 µm).

Parasite sequestration or the accumulation of mature parasite forms in deep vascular beds causes microcirculatory obstruction and has been associated to severe malaria in adults and children with *P. falciparum* [reviewed in^28^]. Parasite sequestration is also an established feature of *P. coatneyi* infection^14–16,19,20^, with sequestered *P. coatneyi* infected RBCs proposed to cytoadhere to similar endothelial molecules as *P. falciparum*^19,22^. Here, we assessed necropsied tissue samples from animals who developed SMA and those who did not for differences in parasite accumulation in deep tissue vasculature. Necropsies were performed on paired animals simultaneously when one animal in the pair developed SMA. The pattern of parasite accumulation in deep vascular beds was similar between both species and did not appear to be associated with any inflammation or tissue damage in either group. The heart was a major site of parasite accumulation in infected rhesus macaques (4 of the 4 rhesus macaques) versus only one of the cynomolgus macaques (Fig. 2A). Mature parasites accumulated within the lumen of small capillaries in the musculature of the right and left ventricles, primarily in the microvasculature. We did not appreciate the same phenomenon in the coronary arteries nor in the great vessels. The heart has also been shown to be a major site of parasite sequestration in children with severe malaria including SMA^29^, in addition to the intestinal tract. However, in our study this may have been due to the higher parasite burden in animals with parasite accumulations in the heart, as all of them (including the one cynomolgus macaque) had parasitaemias above 2.3% the day before necropsy whereas animals without had parasitaemias of less than 1% the day prior to necropsy.

Organs were also assessed for the presence of parasite pigment (haemozoin). The spleen (Fig. 2C,D) and liver were common sites where haemozoin pigment was abundant in both infected species (Fig. 2B). The kidney and lung, and the brain to a lesser degree (3 of 4 animals), were also the major sites of haemozoin deposition in rhesus macaques (4 of 4 animals), and to a lesser extent in the cynomolgus macaques (1 of 4 animals). However, when detected in animal organs, the amount of haemozoin pigment was fairly similar between species. The vast majority of haemozoin in the spleen was within the cytoplasm of macrophages (Fig. 2E,F) and minimally associated with neutrophils (Fig. 2G,H) in both species. As the amount of pigment in the spleens of animals in both species appeared to be similar, we speculate that the haemolytic differences between the two models were not due to extravascular haemolysis of infected RBCs. Future studies can further evaluate this hypothesis by assessing spleen size and pigment quantification in greater detail.

The bone marrow was assessed in 2 animals from both species that yielded adequate samples. Perls’ Prussian Blue stain was used to differentiate haemosiderin iron storage from haemozoin in the bone marrow of infected animals. Although there was no evidence of parasite accumulation in the bone marrow of either species, haemozoin was observed in the bone marrow of both infected rhesus macaques, but only in one of the two cynomolgus macaques whose bone marrows were assessed (Supplementary Table S2). Bone marrow haemozoin deposition is also observed in children with SMA^30^, but due to the limited number of animals in our study this evaluation would need to be repeated with more animals to confirm its significance.

### Features differentiating models of moderate and severe malarial anaemia

Prior to infection with *P. coatneyi*, both macaque species had largely comparable haematological and iron parameters (Supplementary Table S3). Of note, haptoglobin levels at baseline were, on average, 29 mg/dL (95% CI: 5, 53) lower in the rhesus macaques compared to the cynomolgus macaques, but as an acute phase protein increased to similar levels (118.9 mg/dL vs 119.9 mg/dL, respectively) at their peak on day 9.

Rhesus macaques infected with blood-stage *P. coatneyi* met criteria for SMA between 11 and 15 days post-infection (Fig. 1F). At the time of SMA in rhesus macaques, the haematocrit (p = 0.0025), haptoglobin (p = 0.0006) and RPI (p = 0.03) were significantly higher in cynomolgus compared to rhesus (Supplementary Table S3). To identify factors that may contribute to the development of SMA in rhesus macaques and discriminate them from those related to moderate anaemia in infected cynomolgus macaques, we compared factors between the two infected species prior to the majority of rhesus macaques suffering the SMA outcome (days 0 to 11 post-infection).

**Figure 3:**
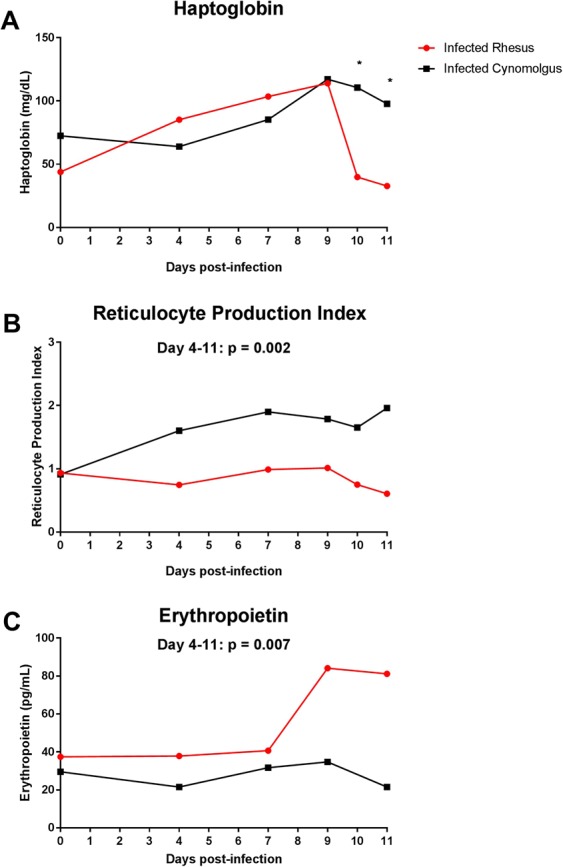
Haematological indicators during early blood-stage *P. coatneyi* infection. (**A**) Haptoglobin, (**B**) reticulocyte production index and (**C**) erythropoietin levels in rhesus (n = 4) and cynomolgus (n = 4) macaques infected with blood-stage *P. coatneyi* parasites. Error bars show SEM for each group. Haptoglobin data were analysed using an unpaired, two-tailed t-test, with *indicating p values < 0.05. Reticulocyte production index and log_10_ erythropoietin were compared between species on days 4 to 11 using linear mixed-effects models that adjusted for linear time trends and day 0 levels, and allowed for individual-specific random effects.

Increased destruction of RBCs (infected and uninfected) is characteristic of malarial anaemia^7,8^, thus we assessed haemolysis in both infected species. The consumption of haptoglobin, a glycoprotein that binds with high affinity to free haemoglobin, can be used to measure intravascular and extravascular haemolysis^31^. When free haemoglobin is present in the plasma, haptoglobin binds rapidly with it forming a complex that is removed by monocytes/ macrophages, resulting in decreased plasma haptoglobin levels [reviewed in^32,33^]. Hypohaptoglobinaemia has been associated with malarial anaemia in African children^34^, and specifically associated with SMA compared to cases of uncomplicated, mild or cerebral malaria^35,36^. In our SMA model, infected rhesus macaques had lower levels of plasma haptoglobin compared to infected cynomolgus macaques on days 10 (50.5 ± 14.56 vs 114.6 ± 18.09 mg/dL; p = 0.03) and 11 (40.72 ± 13.1 mg/dL vs 98.95 ± 9.431 mg/dL; p = 0.01) post-infection (Fig. 3A). The decline in plasma concentrations of haptoglobin in infected rhesus macaques coincided with the drop in haematocrit, whereas infected cynomolgus macaques did not have a comparable decline in their haptoglobin levels. At euthanasia, haptoglobin levels in cynomolgus ranged from 48.8 mg/dL to 93.3 mg/dL whereas in rhesus were < 10 mg/dL to 14.9 mg/dL (p = 0.0006). This suggests that infected rhesus experience exacerbated haemolysis compared to infected cynomolgus macaques, which likely contributes to their severe outcome.

An inappropriately low erythropoietic response is also a key feature of SMA in children. This can be measured by the reticulocyte production index (RPI), which corrects the reticulocyte count for the level of anaemia and thus indicates whether the bone marrow is adequately compensating for the degree of anaemia^36^. An RPI of >3 is indicative of an appropriate bone marrow response to anaemia, whereas an RPI <2 is indicative of a suppressed erythropoietic response [^37^; reviewed in^38^]. In holoendemic regions, a low RPI is characteristic of malarial anaemia, particularly SMA^37,39^. The mean RPI of both macaque species remained below 2 throughout the infection indicating an inadequate bone marrow response to anaemia (Fig. 3B). However, the RPI of cynomolgus macaques was significantly higher than rhesus macaques (p = 0.002; Fig. 3B), with 3 of the 4 cynomolgus macaques having RPI values above 2 during the course of infection, whereas the RPI values of all 4 infected rhesus macaques remained below 2 throughout infection. Thus, rhesus macaques appeared to display a greater defect in erythropoiesis during infection and specifically failed to mount an appropriate erythropoietic response to severe anaemia. To determine whether this inadequate erythropoietic response was due to a deficiency in erythropoietin (EPO), we quantified plasma EPO levels and found that levels were significantly higher in rhesus macaques compared to cynomolgus macaques during infection (p = 0.007; Fig. 3C). Thus, despite higher levels of EPO rhesus macaques failed to mount appropriate reticulocyte responses, as has also been seen in the children with SMA^40,41^. Our results, as well as results from others^25^, suggest that EPO production is not a contributing factor to the inadequate erythropoietic response seen in this model.

### Immunopathogenesis of severe malarial anaemia

We examined levels of immunological markers at baseline and throughout the course of infection that might contribute to disease sequelae. Prior to infection, both macaque species had largely comparable immunological parameters (Supplementary Table S4) with a few exceptions. Of note, the geometric mean of the median fluorescence intensity (MFI) of CD35 at baseline was, on average, 1563 MFI (95% CI: 393, 2732) lower in the rhesus macaques compared to the cynomolgus macaques. Interleukin (IL)-1β levels were below the limit of detection for all cynomolgus macaques, whereas levels were detectable in three of the four rhesus macaques prior to infection. At the time of SMA in rhesus macaques, CD35 expression on RBCs (p = 0.0005), MIP-1α (p = 0.005), IL-23 (p = 0.03), MCP-1 (p = 0.02) and TNF-α:IL-10 ratio was significantly different between the two infected species (Supplementary Table S4).

**Figure 4:**
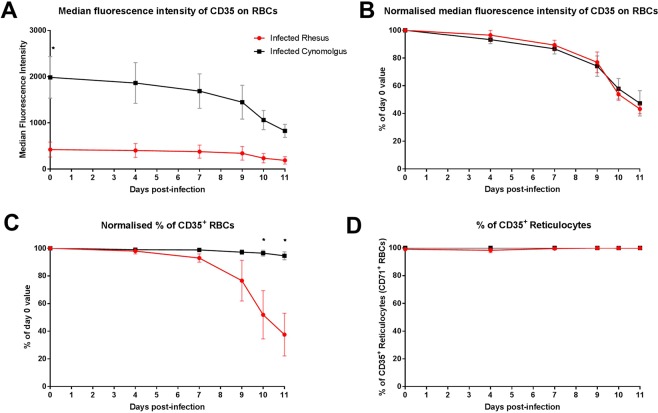
Complement regulatory protein (CD35) on RBCs during the early course of *P. coatneyi* blood-stage infection. Blood samples were collected and assessed during blood-stage *P. coatneyi* infection in rhesus (n = 4) and cynomolgus (n = 4) macaques for (**A**) the median fluorescence intensity (MFI) of RBCs, (**B**) the MFI normalised to the baseline of each animal, (**C**) the percentage of CD35^+^ RBCs normalised to the baseline of each animal and (**D**) the percentage of CD35^+^ reticulocytes (CD71^+^). Error bars show SEM for each group. Data in graph (**C**) were analysed using an unpaired, two-tailed t-test, with *indicating p values < 0.05.

Complement is a component of the innate immune response, and is activated during malaria infection^42^. Complement deposition marks cells for phagocytic destruction and removal by the reticuloendothelial system, while surface complement regulatory proteins protect from complement attack and lysis. Lower levels of complement regulatory proteins, such as CD35, on the surface of RBCs have been observed in children with SMA^11–13^. As noted above, CD35 expression on RBCs was markedly different at baseline between our two models of malarial anaemia (p = 0.01; Supplementary Table S4 & Fig. 4A). However, when normalised to baseline, the proportional loss of CD35 on RBCs during the course of infection did not discriminate the two models (p = 0.496; Fig. 4B). Instead, owing to their low baseline CD35 levels, the overall reduction in the percentage of CD35^+^ RBCs was significant in infected rhesus (59.7 ± 15.8% reduction by day 11; p = 0.014) compared to infected cynomolgus macaques (4.8 ± 2.8% reduction) (Fig. 4C). The percentage of reticulocytes (CD71^+^ RBCs) that were CD35^+^ remained consistent throughout infection in both species (Fig. 4D), indicating that there was no alteration of CD35^+^ immature RBCs produced by the bone marrow during SMA in rhesus macaques, and that the loss of CD35 occurred in circulating normocytes.

**Figure 5:**
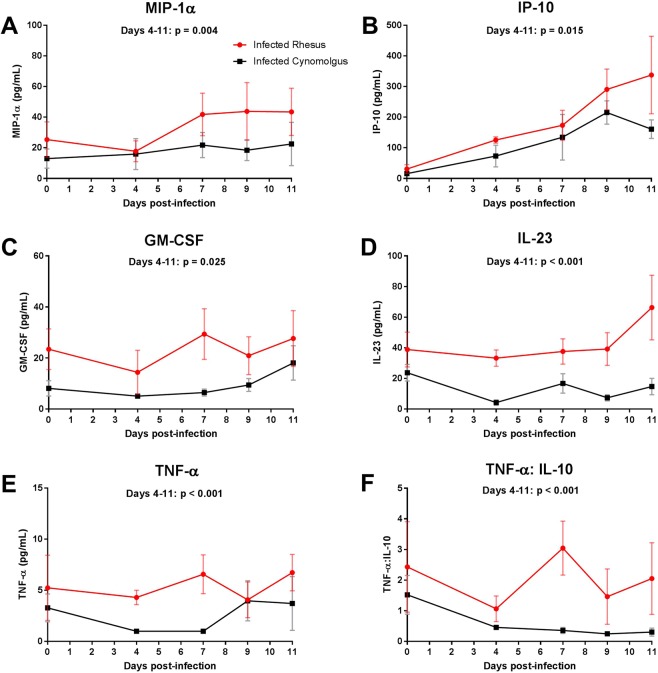
Cytokine and chemokine response during early blood-stage *P. coatneyi* infection in rhesus and cynomolgus macaques. Plasma samples were collected from rhesus and cynomolgus macaques on day 0, 4, 7, 9 and 11 post blood-stage *P. coatneyi* infection and were assessed for (**A**) MIP-1α, (**B**) IP-10, (**C**) GM-CSF, (**D**) IL-23, (**E**) TNFα and (**F**) TNFα: IL-10 ratio levels. Error bars show SEM for each group. Log_10_ biomarker levels were compared between species on days 4 to 11 using linear mixed-effects models that adjusted for linear time trends and day 0 levels, and allowed for individual-specific random effects.

The severity of clinical disease experienced by *Plasmodium* infected individuals can be influenced by the ability to regulate and control their immune responses [reviewed in^43^]. Cytokines and chemokines have a key role in maintaining a balanced response to infection. While an early and robust inflammatory response is critical for controlling acute blood-stage infection, uncontrolled inflammation might contribute to severe malaria pathogenesis including SMA^44–46^. Plasma cytokine and chemokine profiles were assessed in both rhesus and cynomolgus macaques infected with blood-stage *P. coatneyi*. A total of 18 cytokines and chemokines were assessed (full list provided in the Methods) based on previous reports on their relevance to anaemia and SMA [reviewed in^10^]. Baseline levels of cytokines and chemokines did not differ significantly between species, other than IL-1β which was elevated in the rhesus macaques. During infection (days 4–11), six cytokines and chemokines (IP-10, MIP-1α, IL-23, GM-CSF, TNF-α and TNF-α: IL-10 ratio) differed significantly between rhesus who suffered SMA and cynomolgus who did not (Fig. 5A–H & Supplementary Table S5). IP-10 (p = 0.015), MIP-1α (p = 0.004), GM-CSF (p = 0.025), IL-23 (p < 0.001), TNF-α (p < 0.001) and TNF-α: IL-10 ratio (p < 0.001) were significantly higher in the plasma of infected rhesus macaques after adjustment for baseline values. Interleukin-23 was significantly higher in rhesus macaques than in cynomolgus macaques, but this difference appeared to be due in part to decreased levels during infection in the plasma of cynomolgus macaques (Fig. 5D).

**Figure 6:**
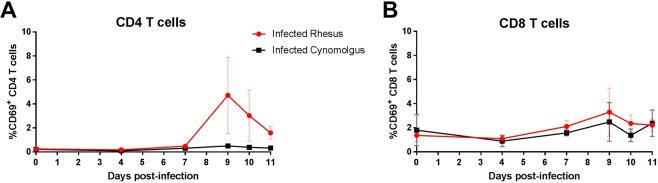
Immune activation of CD4 and CD8 T cells during early blood-stage *P. coatneyi* infection in rhesus and cynomolgus macaques. Blood samples were collected from rhesus and cynomolgus macaques on day 0, 4, 7, 9 and 11 post blood-stage *P. coatneyi* infection. The percentage of (**A**) CD4 and (**B**) CD8 T cells expressing CD69. Error bars show SEM for each group.

T cells have been implicated in the pathogenesis of SMA^47,48^. Thus, we assessed the immune activation of CD4^+^ and CD8^+^ T cells during development of SMA, using a marker of early activation (CD69). There was no significant difference in early activation of CD8^+^ T cells (p = 0.360; Fig. 6B). Although not significantly different, rhesus macaques had a distinct elevation in their activated CD4 T cells at day 9 post-infection (p = 0.174; Fig. 6A) and approached significance at the time of SMA in rhesus macaques (p = 0.08; Supplementary Table S4).

## Discussion

Blood-stage *P. coatneyi* infection in rhesus macaques has previously been described as a relevant model of *P. falciparum* infection in humans, due to its tertian periodicity, and its ability to sequester and to cause severe malaria in these animals^15,19,20,22^. Here, we described the characteristic features of severe malaria anaemia (SMA) using two closely related macaques and found that these closely paralleled features that have previously been described in African children with SMA. We further interrogated the differential expression and temporal patterns of factors previously implicated in SMA in human studies, to support their causal roles in the pathogenesis of SMA using rhesus versus cynomolgus macaques.

In previous *P. coatneyi* infection studies, parameters of malaria-infected animals have been compared to those of uninfected control animals^19,25,49^. In the current study, we compared the parameters and outcomes of *P. coatneyi* blood-stage infection in two closely related host species^50^, the rhesus and cynomolgus macaque, to discriminate pathogenic factors that associate with susceptibility and that appear before the outcome of SMA. Many of the initial studies of blood-stage *P. coatneyi* infection in rhesus macaques suggested it may be a model to study cerebral malaria^16,22,24^ and multi-organ dysfunction;^25^ however in the current study, blood-stage *P. coatneyi* infection in female rhesus and cynomolgus did not cause convulsions, prostration, hyperparasitaemia, jaundice, renal impairment, hypoglycaemia or thrombocytopenia. The single severe outcome of SMA (haematocrit <20%) occurred in *P. coatneyi*-infected rhesus macaques but not cynomolgus macaques. Studies in children have demonstrated that severe malaria most commonly presents as a single condition, such as convulsions or severe anaemia^51–53^. Additionally, in our studies to date, including this report, SMA has been a consistent feature of blood-stage *P. coatneyi* infection in rhesus macaques irrespective of their ages, which have ranged from 4–12 years old. It should be noted that in the earlier animal studies many of the animals had hyperparasitaemia (parasitaemia >10%)^16,22,24^, unlike the current study, thus that higher parasite burden may have contributed to the development of cerebral malaria. Other differences seen between the current study and those previously reported (some also using Hackeri strain of *P. coatneyi*) may be due to differences in factors such as geographic origin and genotype of the rhesus, or diet and other environmental factors.

In the approximately 2-week timeframe of observation in the current study, infected cynomolgus macaques developed mild to moderate anaemia. However, we cannot exclude that cynomolgus macaques may have developed SMA if monitored over a longer period, as has been seen in some but not all infected cynomolgus in a previous report^54^.

One key parameter distinguishing our models of SMA and moderate anaemia was rapid haemolysis, as assessed by an accelerated drop in plasma haptoglobin levels in infected rhesus compared to infected cynomolgus macaques. This drop in haptoglobin occurred at the inflection point of a decline in haematocrit in infected rhesus macaques. In a field setting; however, plasma haptoglobin cannot be used to determine the severity of haemolysis, due to its rapid and often complete consumption during episodes of haemolysis^55^. Haemopexin determinations are recommended to judge the severity of haemolysis in the absence of haptoglobin^55^. We were unable to determine whether the haemolysis was largely intravascular or extravascular. However, using bilirubin as the marker of extravascular haemolysis, as has previously been described [reviewed in^56^], there was no difference in this marker between the two species, suggesting that there were differences in intravascular rather than extravascular haemolysis in the two models. Using parasite pigment deposition in the spleen as a crude measurement of extravascular haemolysis of infected RBCs showed no difference in the presence of haemozoin, suggesting that there was no difference between the clearance of infected RBCs between the two models. In addition, the parasite burden from day 0 to 11 post-infection was comparable between the two models. Taken together these data indicate that there is likely to be accelerated clearance of uninfected RBCs in animals who suffer SMA compared to those that do not. Moreno *et al*.^25^ demonstrated that there was accelerated clearance of biotinylated RBCs in rhesus macaques infected with blood-stage *P. coatneyi*. Similar to our study, they also observed an acute drop in haematocrit that occurred at relatively low parasitaemia, which suggests the destruction of uninfected as well as infected RBCs^25^, similar to humans in whom the vast majority (approximately 90%) of cleared RBCs are uninfected^7,8^. The basis for this is not established, although RBC deformability is reduced during malaria infection, affecting both infected^57,58^ and uninfected RBCs^59,60^, and this is positively associated with the degree of anaemia^60^. Further, it has been suggested^7^ that during acute malaria there is a reduced splenic threshold for rigid^61^ and antibody-coated RBCs^62,63^ leading to removal of RBCs.

In Gambian children, the sequestered parasitic biomass, measured by plasma levels of the secreted parasite protein HRP2, was higher in patients with SMA than uncomplicated malaria^64^. Here, we assessed parasite burden within specific organs and found that mature parasite accumulations within cardiac microvessels was a consistent feature of rhesus who had developed SMA. However, in this small study, this phenomenon could not be dissociated from higher peripheral blood parasite density the day prior to tissue sampling.

Both macaque species supported similar peripheral parasite densities prior to day 12 post-infection, at which point differences in parasitaemia were observed and may have contributed to differences in RBC loss between the two infected species. However, the different trajectories of declining haematocrit in the two species appeared prior to day 12 and before differences in the parasitaemia occurred. Furthermore, the cumulative loss of RBCs exceeded the cumulative level of infected red cells during infection. This suggests that other factors are involved earlier in infection and may contribute to the development of SMA.

Complement regulatory proteins, such as CD35, on the surface of cells protect from attack and lytic effects of complement. CD35 binds to immune complexes, and both can be removed from the RBC surface by macrophages during passage through the reticuloendothelial system^65^. This can leave the RBC vulnerable to complement attack and haemolysis. Levels of CD35, and other complement regulatory proteins, such as CD55 and CD59, are lower on the surface of RBCs in patients with SMA^11–13^. RBCs from these patients also have increased susceptibility to phagocytosis versus those from patients with uncomplicated malaria^13^. While CD35 is expressed as the full-length protein on human RBCs, smaller as well as full length versions of CD35 are expressed on RBCs from non-human primates [reviewed in^66^]. Our assessment of CD35 levels on RBCs by flow cytometry revealed that at baseline levels were substantially lower in rhesus than cynomolgus macaques, and we hypothesise that this could be integral to the susceptibility of these rhesus macaques to develop SMA. Of note, early flow cytometry experiments showed that the E11 clone used in our study bound to CD35 on the surface of RBCs from both rhesus and cynomolgus macaques to a similar degree^67^. The differences between our RBC surface reactivity data for rhesus and those from the previous study may reflect a difference in the origins of the macaques. Rhesus macaques inhabit a wide geographical area^68^, which may have allowed for genotypical differences^69^, and possible differences in CD35 expression patterns on RBCs.

We hypothesise that baseline differences in CD35 levels between children at the time of infection might influence their susceptibility to SMA; studies have assessed expression of complement regulatory proteins at SMA diagnosis and after treatment^11,13^, but not at baseline. After blood transfusion and treatment with anti-malarial drugs for SMA, CD35 recovers to levels comparable to those in children treated for uncomplicated malaria^11,13^, but a potential transient correction due to the presence of transfused RBCs cannot be excluded^13^. Here, the proportional loss of CD35 on RBCs was comparable between the two models, but this led to a significant reduction of CD35^+^ RBCs only in rhesus, which notably had lower baseline CD35 surface levels. Our assessment of CD35 entailed flow cytometry analysis of RBCs after antibody labelling of surface antigens; protein assessment by western blot or mass spectrometry in future studies may reveal further information about protein abundance and variants.

A study of Gabonese children demonstrated an inverse relationship between haematocrit and plasma EPO^40^. As haematocrit decreased, EPO levels increased exponentially^40^. Thus, it is not a reduction in EPO production that contributes to the pathogenesis of malarial anaemia^40^. Similar to these findings in children, and as has previously been shown^25^, rhesus macaques infected with *P. coatneyi* produce EPO in response to the acute anaemia and it is not the absence of EPO that contributes to the inappropriate reticulocyte production in this model^25^. Although erythropoiesis was stimulated (i.e. as indicated by production of EPO) in animals who developed SMA, these animals failed to produce an appropriate reticulocyte response. If there is a block in maturation or release of RBCs, intensive EPO stimulation could lead to accumulation of immature RBCs in the bone marrow^41^. A study of Ghanaian children demonstrated that EPO is increased during SMA to induce erythropoiesis but that maturation or release of reticulocytes is limited/ blocked until after the clearance of parasites^41^. The cause of this reversible suppression of erythropoiesis may be morphological changes in the bone marrow^4,6,70,71^, a direct inhibitory or blockage effect of the parasites^71^, inflammatory mediators^72^ or haemozoin pigment^30,73^. Although our study only assessed a limited number of bone marrow samples from infected animals and needs to be repeated, haemozoin was more frequently observed in the bone marrow of rhesus macaques. The presence of haemozoin has been shown to suppress erythropoiesis and to associate with an inflammatory response *in vitro*^30,74^.

Assessment of peripheral cytokine and chemokine levels demonstrated a more robust pro-inflammatory profile in infected rhesus macaques than cynomolgus macaques, which may contribute to the outcome of SMA in rhesus macaques. Many studies from malaria endemic countries demonstrated an association of a high TNF-α and IL-10 plasma ratios in young children presenting with SMA^44,46,75^, which was also a distinguishing factor between our models of severe and moderate anaemia. Our data suggest that higher levels of TNF- α between days 4 and 11 post-infection were responsible for this imbalance, whereas a number of field studies attribute this imbalance to lower levels of IL-10 in SMA rather than an increase in TNF-α^46,75^. In either case, SMA was associated to a dysregulated inflammatory response. IL-23 was also a distinguishing factor of our models of moderate and severe anaemia; however, a study in Kenyan children showed IL-23 was elevated in children with malarial anaemia, but did not differentiate children with SMA and those with mild malarial anaemia^76^. At the time of SMA in rhesus macaques, GM-CSF and IP-10 did not discriminate SMA from moderate anaemia, as has been reported in children presenting at hospital with SMA^77^. However, our assessment during the course of infection highlighted that higher levels of GM-CSF and IP-10, as well as MIP-1α, in rhesus may be important in the development of SMA. GM-CSF is known to promote erythropoiesis [reviewed in^78^] and in *P. falciparum* infection it can synergise with TNF-α to increase the anti-parasitic activity of neutrophils during blood-stage infection^79^. Plasma levels of IP-10 have been associated to cerebral malaria^80^ and increased levels of MIP-1α have been observed in children with severe malaria^81^, but no such associations has been made specifically with SMA. Our data suggest that activation of CD8^+^ T cells, as assessed by CD69, does not play a role in the development of SMA during acute infection, with the caveat that peripheral blood measurements may not reflect changes occurring within tissue. Although not statistically significant, an increase in activated CD4^+^ T cells in rhesus macaques coincided with the drops in haematocrit and haptoglobin levels. Thus, the role of CD4^+^ T cells in the development of SMA may still be important. Additionally, activation of innate immune cells, such as monocytes and neutrophils, may have a more important role during acute malaria^82–85^.

This study adds to the accumulating body of evidence^25,86^ that blood-stage *P. coatneyi* infection in rhesus macaques is a relevant and valuable model for investigating SMA, and supports the causal role for several factors previously associated with SMA in children. *P. coatneyi* infected rhesus macaques suffer SMA with rapid onset and features including haemolysis and inadequate reticulocyte production, similar to children. As in children, increased EPO expression had no clear impact on reticulocyte levels in the blood of rhesus macaques. Increased pro-inflammatory cytokine responses [reviewed in^10^] and low levels of CD35 on RBCs^11–13^ have been seen in children at the time of SMA. Our data showing increased pro-inflammatory responses during early infection and low levels of CD35 on RBCs at baseline are consistent with the idea that these have causal roles in the pathogenesis of SMA. The findings from this study highlight the relevance of *P. coatneyi* blood-stage infection in rhesus macaques as a suitable model to further investigate SMA. Future studies of interventions that modulate host inflammatory responses or CD35 levels on RBCs in *P. coatneyi* infected rhesus macaques may identify new therapeutic approaches for SMA.

## Supplementary information

Supplementary Information
