# Supplementary material for: A primate model of severe malarial anaemia: a comparative pathogenesis study
Source: Sci Rep. 2019 Dec 12;9:18965. doi: 10.1038/s41598-019-55377-3 (PMC6908728; doi:10.1038/s41598-019-55377-3)
Supplement: Supplementary file 1 — Supplementary Information [file 41598_2019_55377_MOESM1_ESM.pdf]

Supplementary Information

Title: A primate model of severe malarial anaemia: a comparative pathogenesis study

Amber I. Raja, Elizabeth B. Brickley, Jessica Taaffe, Timmy Ton, Zhen Zhao, Kevin W. Bock, Sachy Orr-Gonzalez, Marvin L. Thomas, III, Lynn E. Lambert, Ian N. Moore, Patrick E. Duffy

Table S1. Severe outcomes for end of study criteria. Paired animals were euthanised when one animal in the pair reached any of the pre-determined severe malaria criterion.

**Severe malaria outcome**	**Indication**
Severe anaemia	A haematocrit drop over 60% from baseline, or less than 20% absolute.
Hyperparasitaemia	15% to 19% for more than 24 hours with other CBC and Chem values not out of normal range.
	Parasitaemia 15% to 19% with other CBC or Chem values out of normal range.
	Parasitaemia over 20%
Cerebral malaria	Any recorded convulsions
Postictal state	Inability to orientate to stimuli
Prostration	Inability to stand or abnormal body posture
Dyspnea	Irregular rhythm or increased respiratory effort when at rest

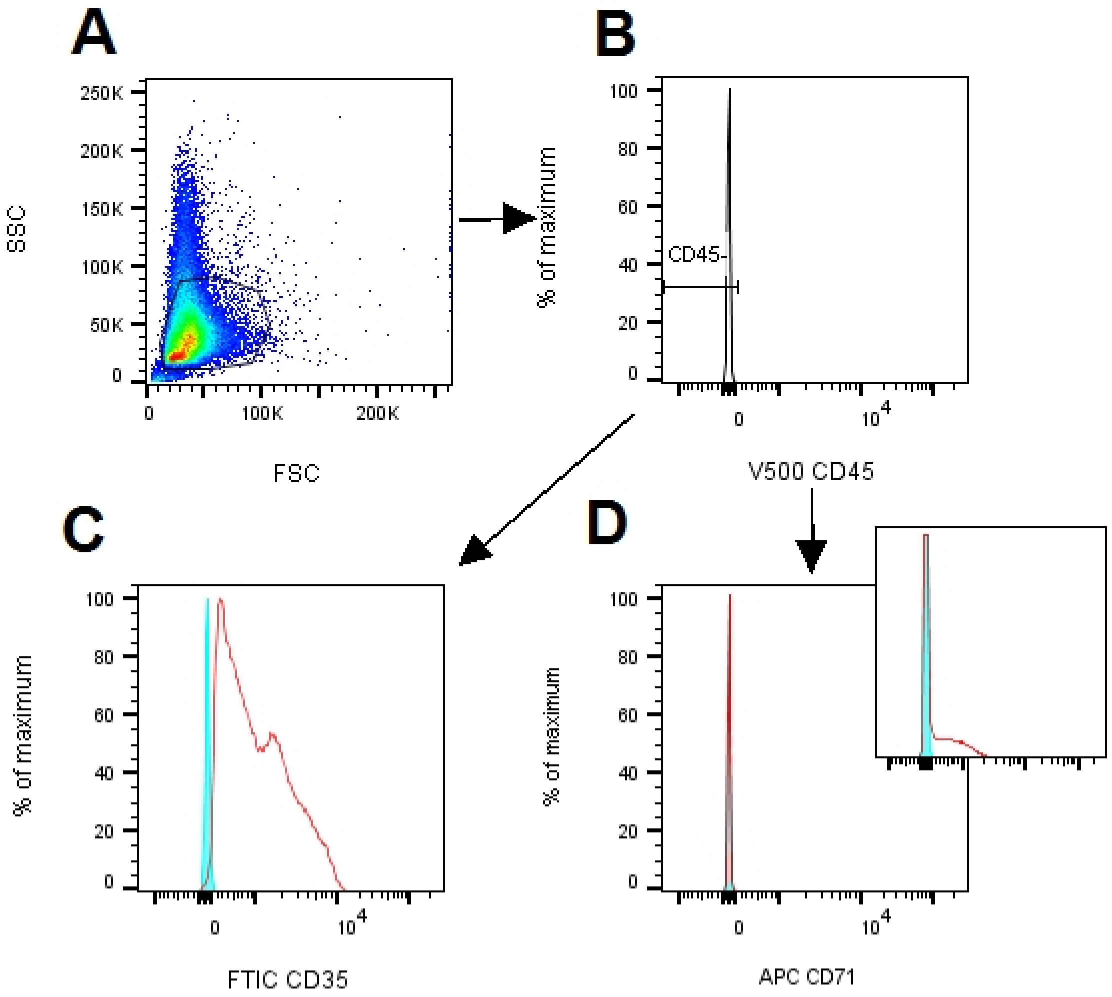

Figure S1. Gating strategy to identify markers on red blood cells. Gating used to identify (**A**) the red blood cell population, (**B**) $CD45^-$, (**C**) $CD35^+$ and (**D**) $CD71^+$ red blood cells. Cells were identified as any fluorescence above that demonstrated by the isotype matched control antibodies (**C**-**D**).

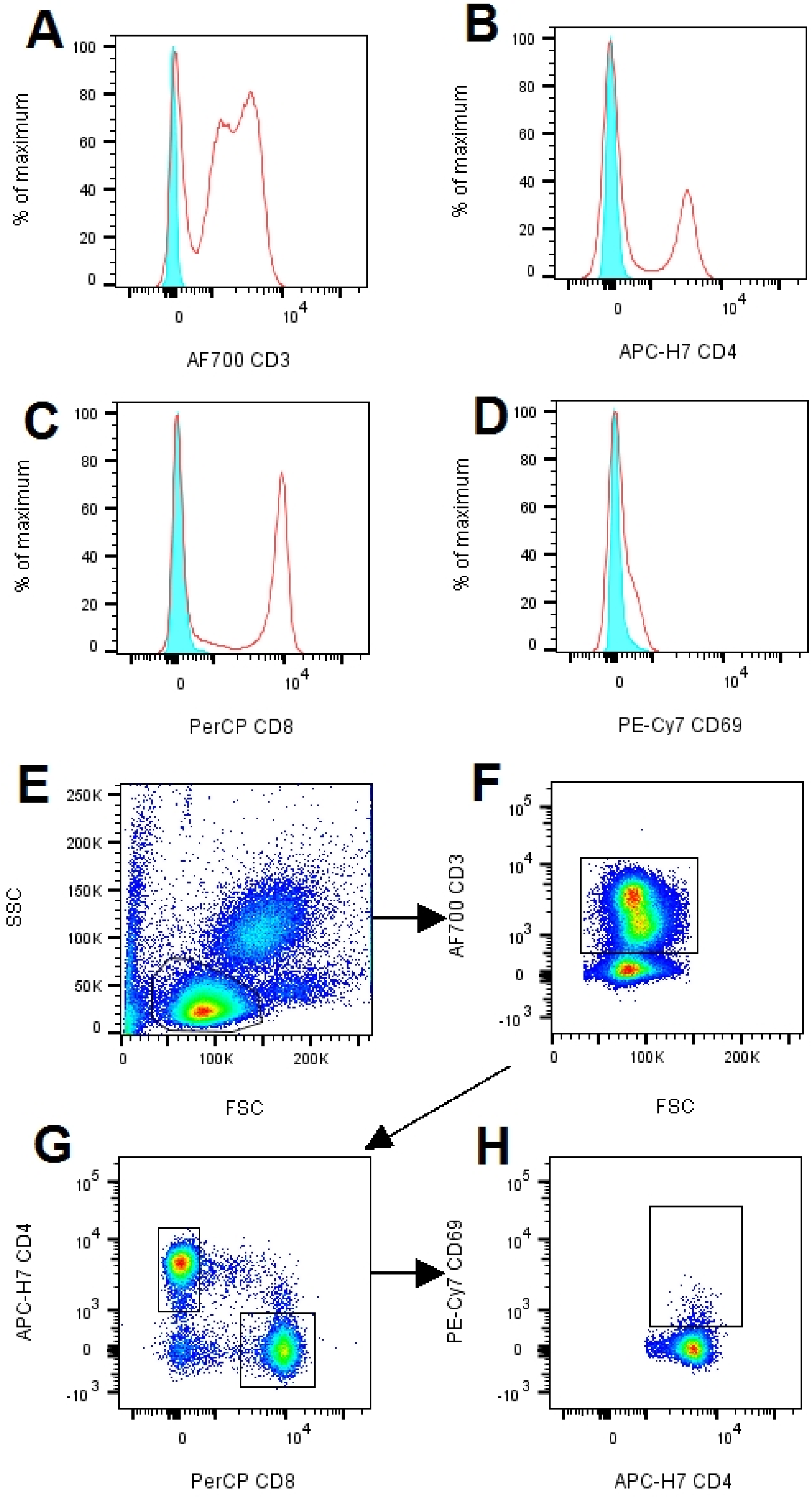
A
% of maximum
AF700 CD3
B
APC-H7 CD4
C
PerCP CD8
D
PE-Cy7 CD69
E
SSC
FSC
F
AF700 CD3
FSC
G
APC-H7 CD4
PerCP CD8
H
PE-Cy7 CD69
APC-H7 CD4

Figure S2. Gating strategy to identify activated circulating CD4$^+$ and CD8$^+$ T cells. Activated CD4$^+$ and CD8$^+$ T cells were identified as any cells with fluorescence above that demonstrated by the isotype matched control antibodies (**A**-**D**). Gating was used to identify (**E**) the lymphocyte population, (**F**) CD3$^+$ T cells, (**G**) CD4$^+$ and CD8$^+$ T cells, activated CD4$^+$ T cells expressing (**H**) CD69. The gating in (**H**) was also applied to CD8$^+$ T cells (not shown).

Table S2. Parasite accumulation and haemozoin deposition in the bone marrow of *P. coatneyi* infected macaques. Bone marrow from euthanised animals was assessed for the presence of parasite accumulation and haemozoin deposits.

**Infected macaque species (number of animals)**	**Parasite accumulation**	**Haemozoin**
Rhesus (2)	0/2	2/2
Cynomolgus (2)	0/2	1/2

Table S3. Haematological and iron characteristics of the study sample at baseline and endpoint.

		Baseline			Endpoint		
		**Cynomolgus**	**Rhesus**	***P* value, t-test**	**Cynomolgus**	**Rhesus**	***P* value, t-test**
		Geometric Mean (95% CI)			Geometric Mean (95% CI)		
**Haematological Markers**	Haematocrit	40 (35, 45)	42 (38, 46)	*0.37*	26 (22, 31)	19 (17, 20)	*0.0025*
	Haptoglobin	72 (51, 103)	44 (32, 61)	*0.03*	77 (47, 127)	11 (8, 15)	*0.0006*
	RPI	0.8 (0.7, 1)	0.9 (0.5, 1.5)	*0.56*	2 (0.7, 4)	0.4 (0.1, 1)	*0.03*
	EPO	30 (16, 55)	37 (6.4, 218)	*0.70*[a]	68 (8, 590)	255 (120, 541)	*0.12*[a]
**Iron Markers**	Iron	133 (93, 189)	148 (106, 206)	*0.49*	129 (36, 469)	107 (61, 187)	*0.40*
	Ferritin	16 (2.7, 99)	12 (3.5, 41)	*0.66*[a]	133 (48, 367)	162 (38, 694)	*0.74*[a]

Abbreviations: RPI, reticulocyte production index; EPO, erythropoietin.

[a]t-tests were performed on the log_{10}-transformed biomarker levels.

Table S4. Immune mediator characteristics of the study sample at baseline and endpoint.

		Baseline			Endpoint		
		Cynomolgus	Rhesus	*P* value, t-test[a]	Cynomolgus	Rhesus	*P* value, t-test[a]
		Geometric Mean (95% CI)			Geometric Mean (95% CI)		
**Complement**	CD35	1746 (612, 4981)	348 (117, 1038)	*0.01*	691 (401, 1190)	98 (47.3, 203)	*0.0005*
**Cytokines & Chemokines**	MIP-1α	10 (3, 36)	17 (3, 105)	*0.48*	6 (2, 22)	46 (22, 96)	*0.005*
	TNF-α	3 (0.7, 10)	3 (0.5, 20)	*0.82*	2 (0.3, 13)	3 (0.6, 16)	*0.55*
	IP-10	15 (7, 30)	22 (5, 98)	*0.46*	125 (75, 209)	184 (74, 448)	*0.29*
	IL-23	22 (10, 50)	32 (8, 121)	*0.48*	12 (4, 34)	45 (16, 127)	*0.03*
	MCP-1	863 (400, 1866)	589 (354, 979)	*0.24*	1557 (805, 1310)	524 (235, 1169)	*0.02*
	MIP-1β	32 (32, 18)	22 (7, 67)	*0.38*	23 (11, 46)	18 (3, 91)	*0.67*
	IFN-γ	2 (2, 2)	2 (2, 2)	*1.00*	2 (1, 2)	2 (0.6, 9)	*0.50*
	IL-1β	2 (2, 2)	7 (2, 5)	*0.03*	3 (1, 7)	3 (1, 8)	*0.91*
	IL-4	7 (3, 15)	5 (3, 10)	*0.41*	3 (1, 8)	5 (1, 21)	*0.43*
	IL-5	4 (4, 4)	4 (4, 4)	*1.00*	4 (4, 4)	4 (4, 4)	*1.00*
	IL-6	3 (0.8, 15)	2 (1, 3)	*0.23*	13 (0.4, 384)	17 (3, 83)	*0.84*
	IL-18	3256 (1971, 5377)	1844 (300, 11351)	*0.37*	2402 (1037, 5564)	1618 (352, 7430)	*0.50*
	IL-10	2 (2, 2)	2 (2, 2)	*1.00*	8 (0.5, 120)	2 (2, 2)	*0.19*
	IL-12p40	13 (13, 13)	13 (13, 13)	*1.00*	13 (13, 13)	13 (13, 13)	*1.00*
	RANTES	1031 (287, 3709)	1039 (115, 9425)	*0.99*	636 (134, 3015)	426 (158, 1148)	*0.51*
	Eotaxin	84 (33, 213)	83 (40, 172)	*0.97*	23 (6, 82)	25 (13, 46)	*0.86*
	G-CSF	30 (10, 93)	43 (6, 297)	*0.64*	25 (10, 64)	36 (12, 111)	*0.47*
	GM-CSF	7 (3, 18)	18 (4, 79)	*0.13*	11 (3, 53)	21 (2, 242)	*0.52*
	TNF-α:IL-10	1 (0.3, 4)	1 (0.2, 9)	*0.82*	0.2 (0.1, 0.8)	1 (0.3, 7)	*0.03*
**T cells**	$CD4^+CD69^+$	0.2 (0.1, 0.6)	0.2 (0.1, 0.6)	*0.60*	0.6 (0.1, 4)	3 (1, 6)	*0.08*
	$CD8^+CD69^+$	0.9 (0.1, 7)	1 (0.4, 3)	*0.74*	0.8 (0.1, 8)	1 (0.3, 6)	*0.57*

Abbreviations: MIP, macrophage inflammatory protein; TNF, tumor necrosis factor; IP-10, interferon-gamma-inducible protein 10; IL, Interleukin; MCP-1, monocyte chemotactic protein-1; IFN-γ, Interferon-gamma; RANTES, regulated on activation, normal T cell expressed and secreted; G-CSF, granulocyte colony-stimulating factor; GM-CSF, granulocyte macrophage colony-stimulating factor.

[a]t-tests were performed on the log_{10}-transformed biomarker levels.

Table S5. Cytokine and chemokine characteristics between days 4 and 11 of infection.

		**Mean Difference** (95% CI)	***P* value**
**Immune Mediators**	MIP-1α	0.5 (0.2, 0.8)	*0.004*
	TNF-α	0.5 (0.3,0.7)	*<0.001*
	IP-10	0.3 (0.1, 0.6)	*0.015*
	IL-23	0.7 (0.5,1)	*<0.001*
	MCP-1	-0.2 (-0.6, 0.2)	*0.23*
	MIP-1β	-0.1 (-0.3, 0.1)	*0.58*
	IFN-γ	0.1 (-0.1, 0.3)	*0.26*
	IL-1β	0.1 (-0.1, 0.3)	*0.20*
	IL-4	-0.04 (-0.3, 0.2)	*0.70*
	IL-5	0 (0,0)	*1.00*
	IL-6	-0.3 (-0.6, 0.1)	*0.18*
	IL-18	0.01 (-0.5, 0.6)	*0.95*
	IL-10	-0.1 (-0.5, 0.2)	*0.46*
	IL-12p40	0.1 (-0.05, 0.2)	*0.23*
	RANTES	-0.1 (-0.3, 0.1)	*0.57*
	Eotaxin	-0.1 (-0.2, 0.1)	*0.48*
	G-CSF	-0.1 (-0.2, 0.1)	*0.42*
	GM-CSF	0.3 (0.04, 0.6)	*0.025*
	TNF-α:IL-10	0.6 (0.3, 0.9)	*<0.001*

Abbreviations: MIP, macrophage inflammatory protein; TNF, tumor necrosis factor; IP-10, interferon-gamma-inducible protein 10; IL, Interleukin; MCP-1, monocyte chemotactic protein-1; IFN-γ, Interferon-gamma; RANTES, regulated on activation, normal T cell expressed and secreted; G-CSF, granulocyte colony-stimulating factor; GM-CSF, granulocyte macrophage colony-stimulating factor.

Mean levels of cytokines and chemokines were compared between infected rhesus and cynomolgus macaques using linear mixed-effects models that adjusted for linear time trends and day 0 levels and allowed for individual-specific random effects.
